# A Treatise on the Epidemic Cholera

**Published:** 1833-01-01

**Authors:** 


### A Treatise on the Epidemic Cholera, as it has prevailed in India, for the Purpose of ascertaining a successful Mode of Treatment; with a Critical Examination of all the Works which have hitherto appeared on the Subject. By *F. Corbyn, Esq.* Bengal Establishment. Calcutta, 1832.

Little did Mr. Corbyn dream, when he finished his volume, that at least 500 authors, to him unknown, had written on cholera about the time that volume was closed ! Mr. Corbyn has given a digest, not quite so full as we have done in this Journal, of the reports made to the three Presidencies, together with analyses of several detached works, which had appeared previously to the appearance of the epidemic in Russia, or in the Western parts of Europe. The information, therefore, to be derived from this volume by the European practitioner *now* is very small—if we except the criticisms of Mr. Corbyn himself. These are, occasionally, tart enough, and the author has expended an unusual share of his critical acumen on the Editor of this Journal, though certainly in a gentlemanly manner, and without any tincture of illiberality. It is chiefly at Dr. Johnson's theoretical speculations that Mr. Corbyn has levelled his censures ; and Dr. J. no doubt, was unaware of the weight of his sins; for, by Mr. Corbyn's account, he has led almost the whole of our Indian brethren astray in matters of theory.

"The prevalence of the Cholera in an Epidemic form has given rise to a number of treatises, in which attempts are made to explain its proximate cause, from which the mode of treatment is necessarily deduced. Before I proceed to a consideration of the principles which the authors of these works have endeavoured to establish, I must take notice of those which form the basis of Dr. James

Johnson's practice, as that gentleman considers the disease as an Endemic, to which the inhabitants of tropical climates are at all times liable. The distinguished author of the Influence of Tropical Climates on European Constitutions, and of the Influence of the Atmosphere on Health, was among the first to attract the public attention to the disease; and, accordingly it is to be supposed, that his views have had considerable influence in forming the opinions, and directing the practice of the medical officers of the three Presidencies." 215.

Again, at page 253, Mr. C. observes :—

"I proceed to examine the Reports furnished by the medical officers at the three Presidencies, commencing with those of Bengal, in which the same train of reasoning with that of Dr. Johnson, has been followed with very little variation."

Once more, he says, at p. 273 :

"Mr. Scot, the Secretary of the Medical Board of the Madras Establishment, on whom has devolved the task of digesting various reports from the Medical Department on that Establishment, unequivocally follows Dr. Johnson's train of reasoning on the proximate cause, and deduces from it the same course of treatment."

If Dr. Johnson has erred in theory or practice, it is evident that he has done so in good company; for he is not vain enough to suppose that the short notice which he took of cholera, from observing a few cases of the disease in a sporadic form, was capable of leading astray such men as Jameson and Scot. Of Mr. Corbyn's own doctrines we cannot form a very clear idea. He seems, like many critics, to be more successful in demolishing the theories of others, than in the establishment of his own. If we understand him rightly, he looks upon cholera as a purely inflammatory disease, and therefore to be combated by depletion and sedatives. The following extract will convey some notion of Mr. Corbyn's therapeutics, and perhaps of his pathology also.

"The indications of cure are, first, to allay the irritability and spasm, and prevent inflammation. Second, to excite biliary effusion into the bowels, and alvine dejections of a healthy consistence. Third, to determine blood to the surface. Fourth, to restore the system to its former tone and healthy action. The first indication is accomplished by bleeding, sedatives, and anti-acids. The second, by calomel and drastic purgatives. The third, by counter-irritants. And the fourth, by corroborants.

First, bleeding. This may be effected by the lancet, by leeches, or by cupping. The most successful is by the lancet; but we find that it is not always practicable; either syncope comes on, or otherwise congestion has taken place, which prevents the flow of blood from the arm or the jugular vein; in either case, we should have recourse without delay to leeches, which ought to be applied over the epigastrium. They are not, however, at all times and in all places to be procured, to the number required. When this is the case, cupping supplies the desideratum.

The extraction of blood from a large vessel, at a distance from the inflamed part, will be more beneficial than topical bleeding. Clinical practice shews, that the patient's safety absolutely depends upon an immediate stop being put to the first approach of inflammation and congestion; and that topical bleeding only proves powerfully efficacious when general plethora or congestion has been previously removed. We shall find, therefore, according to the urgency and importance of the case, that venesection, or arteriotomy will not only be once, but repeatedly necessary, in conjunction with leeches and cupping. The quantity of blood to be taken away must depend, in like manner, upon the severity and urgency of the symptoms, the strength, and age of the patient. We must ever consider, however, that in the majority of cases, especially among Europeans, cure depends upon the boldest use of the lancet. In the records to be found in the second part of this work, instances are stated in which life seems to have been saved by the sacrifice of some hundred ounces of blood in a few days; and that 60 ounces were drawn at a time, with unequivocal success, in which the small extractions would have failed to render the least relief.

Our object, in this disease, is obviously to evacuate a considerable quantity of blood; hence we must operate in such a manner as not to induce fainting. Now we know from experience, that during the operation of evacuating blood, the patient is liable to fall into a state of syncope; a horizontal position, therefore, is to be preferred to any other; for fainting is not so liable to occur in a horizontal as in an erect posture. But admitting that in any position fainting occurs, we should not on that account suppose the blood will not again flow, we must wait patiently, at the same time moving the arm in all the variety of positions that can probably assist in bringing the openings of the skin and other integuments to correspond with that of the vein, until the blood begins to flow. Throwing the muscles of the part into constant action, or giving the patient a cane or other firm substance to turn frequently around in the hand, will often produce a flow of blood which would not otherwise ensue. The experienced practitioner knows these circumstances to be true; but as the tyro, ignorant of such facts, might not adopt these alternatives, I have thought it proper to be thus particular.

With respect to arteriotomy, we are aware that its most strenuous friends have shrunk from any attempt of this kind on the larger arteries. Perhaps in no affection is arteriotomy in the smaller branches more desirable than in Epidemic Cholera, in which we require immediate and large evacuations of blood. But let us now suppose that we fail to obtain blood by venesection, arteriotomy, and cupping; we must then have recourse to such measures as will draw the blood to the surface, such as friction and the hot bath. It often happens, that we can draw blood while the patient is in the hot bath, which would fail to flow in any other situation. The bath must therefore be at hand.

Sedatives are laudanum, calomel, magnesia, and hot bath.

Laudanum. This medicine may be given in doses of sixty to eighty and one hundred and fifty drops with great safety, and the effect is favourable in producing sound sleep, assuaging pain, removing spasm and irritability. But laudanum in less doses operates in a different way; fifteen and twenty to thirty and forty drops produce a high degree of excitement, sometimes apparent inebriety and delirium, and if the patient should doze, frightful dreams awake him in convulsive startings.

Calomel, in doses of from fifteen to twenty grains, is a sedative, and has the singular good qualities of immediately stopping violent vomiting and purging, removing spasmodic irritability, producing tranquillity of mind, exciting the secretion of the liver, and preventing the progress of inflammation. I have known a patient labouring under frequent dysenteric evacuations, with tenesmus, to be under the common course of small doses of calomel and opium for a fortnight without effect, and strange to say, one dose of twenty grains of calomel, at once stopped the purging, removed the tenesmus, and soon restored the bowels to their former tone. Calomel, in doses of from one to five and ten grains, acts as a stimulant, produces vomiting, and violent purging, lassitude, restlessness, and almost insupportable griping pains of the abdomen. Although such are the effects of calomel on the constitution, it is equally important to mention the form in which calomel ought to be given in acute diseases." 197.

From the above extract it will be seen that Mr. Corbyn does not differ, in any essential manner, from the practice which Dr. Johnson suggested long before the disease broke out in its virulent and epidemic form in the East. Dr. Johnson would say, that Mr. Corbyn has misunderstood, rather than unfairly criticised his theoretical speculations, and freely absolves him from any intention to misrepresent him. It is now too late in the day to quarrel about hypotheses, since a wide field, unfortunately, for observation in our own country, has tended to cool the most ardent of our theorists, and compelled them to acknowledge their ignorance.

We shall advert to a circumstance or two before we close our notice of this volume. The following passage at page 45, will set at rest the assertion that the epidemic is a new disease of 1817. Mr. Corbyn, who has been in India since 1814, and constantly among cholera patients, must surely be allowed to be a competent judge on the present occasion.

"In 1814, I was myself eye-witness to the destructive operation of this disease on board the ship *Mangles*, on which I embarked for India. We had been at sea about two months, when it burst forth with awful violence. During the short period of six weeks, sixty-four bodies were thrown overboard, and four men died one after another just as we had cast anchor in Table Bay." 45.

Thus then a ship from England, three years before the outbreak in Jessore, presented a frightful visitation of cholera, two months after she left an English port, and buried 68 men between England and the Cape!

Mr. Corbyn comes to the conclusion, from all that he has seen of Cholera, that it is not contagious.

"From personal experience, I feel satisfied that the notion of the Cholera being contagious is quite unfounded. I breathed the atmosphere of my hospital in the centre division of the grand army, where were the most distressed and the poorest classes of people, morning, noon, and night; yet neither myself, nor any of the attendants were affected. During this time I have also visited officers on the staff whose tents were adjacent, but I never communicated the disease to any.

Those, however, who have written on contagion, advance, that some constitutions are not susceptible of contagion in any way, yet they may become the medium of communicating it to others. We ought to be cautious in maintaining opinions which suppose contagion in so remote and equivocal a manner as this, knowing the alarming consequences of such a belief. Through fear of contagion the sick may be forsaken and die for want of proper care; hospitals may become crowded, and cleanliness neglected for want of attendants, and thus a diseased atmosphere induced." 97.

We shall only make one more observation on the treatment which Mr. Corbyn has laid down in the extract already quoted. He makes no allusion whatever to the premonitory diarrhœa—makes no provision for its cure—a convincing proof, we imagine, that the said premonitory diarrhœa made no conspicuous figure in the character of the disease in India, else it would not thus be overlooked. This is the grand distinction between the disease as it appeared in the Eastern and in the Western hemisphere. Here it forms a most important beacon or warning, by attention to which nine-tenths of the cases of fatal cholera would be prevented.

Mr. Corbyn's object was to present the young Indian practitioner with a digest of what had been written on cholera, up to the year 1831 or 1832, so as to form a portable book of reference. The publication will answer the purpose for which it was designed, but, for the reasons already stated, its interest in this country is much diminished by our unfortunate familiarity with the complaint.

